# Quality of life, tuberculosis and treatment outcome; a case–control and nested cohort study

**DOI:** 10.1183/13993003.00495-2019

**Published:** 2020-08-06

**Authors:** Sumona Datta, Robert H. Gilman, Rosario Montoya, Luz Quevedo Cruz, Teresa Valencia, Doug Huff, Matthew J. Saunders, Carlton A. Evans

**Affiliations:** 1 Dept of Infectious Disease, Imperial College London, London, UK; 2 IFHAD: Innovation for Health and Development, Laboratory for Research and Development, Universidad Peruana Cayetano Heredia, Lima, Peru; 3 IPSYD: Innovacion Por la Salud Y el Desarollo, Asociación Benéfica Prisma, Lima, Peru; 4 Dept of International Health, Johns Hopkins Bloomberg School of Public Health, Baltimore, MD, USA

## Abstract

**Background:**

Global tuberculosis policy increasingly emphasises broad tuberculosis impacts and highlights the lack of evidence concerning tuberculosis-related quality of life (QOL).

**Methods:**

Participants were recruited in 32 Peruvian communities between July 13, 2016 and February 24, 2018 and followed-up until November 8, 2019. Inclusion criteria were age ≥15 years for “patients” (n=1545) starting treatment for tuberculosis disease in health centres; “contacts” (n=3180) who shared a patient's household for ≥6 h·week^−1^; and randomly selected “controls” (n=277). The EUROHIS-QOL questionnaire quantified satisfaction with QOL, health, energy, activities of daily living (ADL), self, relationships, money and living place.

**Findings:**

Newly diagnosed tuberculosis was most strongly associated with lower QOL scores (p<0.001). Patients initially had lower QOL than controls for all EUROHIS-QOL questions (p≤0.01), especially concerning health, ADL and self. Lower initial QOL in patients predicted adverse treatment outcomes and scores <13 points had 4.2-fold (95% CI 2.3–7.6) increased risk of death *versus* those with higher QOL scores (both p<0.001). Patient QOL was re-assessed 6 months later, and for patients with successful treatment QOL became similar to participants who had never had tuberculosis, whereas patients who did not complete treatment continued to have low QOL (p<0.001). Multidrug-resistant tuberculosis was associated with lower QOL before and during treatment (both p<0.001). Contacts had lower QOL if they lived with a patient who had low QOL score (p<0.0001) or were a caregiver for the patient (p<0.001).

**Conclusions:**

Tuberculosis was associated with impaired psychosocioeconomic QOL which recovered with successful treatment. Low QOL scores predicted adverse treatment outcome. This brief EUROHIS-QOL eight-item questionnaire quantified the holistic needs of tuberculosis-affected people, potentially guiding patient-centred care.

## Abstract 2

**The brief EUROHIS quality-of-life questionnaire can be applied to assist in providing holistic, personalised care to TB-affected families, and guide multisystem interventions required to improve their wellbeing and TB treatment outcome** https://bit.ly/2VhDKFM

## Introduction

Tuberculosis (TB) makes ∼10 million people ill each year globally, and kills 1.5 million of them, more than any other infection [1]. A TB diagnosis has consequences far beyond patients' physical symptoms, including associations with mental health and catastrophic costs [2, 3]. The World Health Organization (WHO) End TB Strategy aims to dramatically reduce TB cases and eliminate economic and social burden through integrated patient-centred care and prevention [4, 5]. With currently available diagnostics and treatment the End TB Strategy goals will not be met. Thus, there is increasing priority to mitigate TB-related financial costs and provide support to the psychological and social consequences of TB complementing the longstanding focus on biomedical interventions.

The WHO defines health as “a state of complete physical, mental and social wellbeing and not merely the absence of disease or infirmity”, yet national TB programmes are almost universally evaluated in terms of the number of people they have treated and cured, never their impact on wellbeing [6]. Economists highlight the importance of populations' wellbeing to improve productivity and economy, and advocate its use as an indicator of societal progress [7, 8]. Tools measuring “quality of life” (QOL) are most appropriate for assessing subjective wellbeing, because they assess satisfaction and functioning that respond to life events and are relatively stable compared to substantially different, fleeting feelings of euphoria or happiness [8].

The WHO strategic and technical advisory group (STAG) for TB in 2017 stated that ignoring QOL in TB is “unacceptable and unethical”, recommending that the WHO pursue assessment of TB-related QOL [9]. However, there is no TB-specific QOL questionnaire available. Existing tools such as the EuroQOL-5D and the 36-item Short Form Health Survey, which are used for quality-adjusted life years calculations, have been used in TB-affected people [10, 11]. However, these tools neglect economic and social domains that are important for the synergistic relationship between TB, poverty and isolation. The WHO QOL group created a 100-item questionnaire that subjectively measures universal aspects of QOL, applicable to different languages and cultural settings. Truncated versions brief enough for operational use while maintaining psychometric properties, like the 24-question WHO-QOL-BREF, and subsequent eight-question EUROHIS-QOL were produced [12]. Both tools assess four dimensions of QOL highlighted to be affected by TB: physical health, psychological health, social interactions and satisfaction with living conditions, including economic QOL [12–16]. The EUROHIS-QOL is brief without losing the psychometric properties of the familiar WHO-QOL-BREF, but has only been used to evaluate QOL in few conditions [12].

We aimed to evaluate the EUROHIS-QOL tool for quantifying QOL in TB-affected people (patients and their contacts) *versus* healthy community controls, and to assess whether QOL at the time of diagnosis predicts treatment outcome, including survival.

## Methods

Ethics approval was given by the following committees: the Peruvian Ministry of Health (DIRESA Callao, Peru), Asociacion Benefica Prisma (Peru) and Imperial College London (UK).

### Participant recruitment

For this case–control study with a nested prospective cohort study, from July 13, 2016 to February 24, 2018 participants were recruited concurrently in 15 desert shantytowns and 17 urban communities in Callao, Peru, selected for their high TB case notification rates, and followed-up until November 8, 2019. Inclusion criteria were age ≥15 years in people starting treatment for TB disease in community health centres (termed “patients”; n=1545); people who reported sharing a patient's household for ≥6 h·week^−1^ during the 2 weeks before a patient of any age in their household commenced TB treatment (termed “contacts”; n=3180); and randomly selected community (termed “controls”; n=277). Controls were selected by allocating a number to each residence in the 32 participating communities, and using a random number generator to select households, who were interviewed unless no adult gave written informed consent (in which case another randomly selected household was invited). We termed contacts who were a patient's parent or spouse “carers”, because they were likely to have a caring role for the patient in their household. We termed other contacts “noncarers”. Exclusion criteria were inability or refusal to give informed written consent/assent.

### Measures

After consent, the EUROHIS-QOL questionnaire (see box 1 for the English version, and supplementary table S2 for the Spanish translation used) was read immediately to participants who chose their responses. For patients recruited after November 18, 2016, this was repeated after 6 months’ treatment. To minimise loss to follow-up, three follow-up visits were attempted for each patient. Details of the EUROHIS-QOL, psychosocial, socioeconomic, demographic, clinical and treatment outcome data collected are shown in box 2.

BOX 1 **The EUROHIS-QOL tool in English.**

**Table:** 

**Please listen to the questions with regards to quality of life and pick the best option for you. We ask that you think about your life in the past 2 weeks.**		
**Questions**	**Options**	**Response**
**1. How would you rate your quality of life?**	A. Very poor	
	B. Poor	
	C. Neither good nor bad	
	D. Good	
	E. Very good	
**2. How satisfied are you with your health?**	A. Very dissatisfied	
	B. Dissatisfied	
	C. Neither satisfied nor dissatisfied	
	D. Satisfied	
	E. Very satisfied	
**3. Do you have enough energy for everyday life?**	A. Not at all	
	B. A little	
	C. Moderately	
	D. Mostly	
	E. Completely	
**4. How satisfied are you with your ability to perform your daily living activities?**	A. Very dissatisfied	
	B. Dissatisfied	
	C. Neither satisfied nor dissatisfied	
	D. Satisfied	
	E. Very satisfied	
**5. How satisfied are you with yourself?**	A. Very dissatisfied	
	B. Dissatisfied	
	C. Neither satisfied nor dissatisfied	
	D. Satisfied	
	E. Very satisfied	
**6. How satisfied are you with your personal relationships?**	A. Very dissatisfied	
	B. Dissatisfied	
	C. Neither satisfied nor dissatisfied	
	D. Satisfied	
	E. Very satisfied	
**7. Have you enough money to meet your needs?**	A. Not at all	
	B. A little	
	C. Moderately	
	D. Mostly	
	E. Completely	
**8. How satisfied are you with the conditions of your living place?**	A. Very dissatisfied	
	B. Dissatisfied	
	C. Neither satisfied nor dissatisfied	
	D. Satisfied	
	E. Very satisfied	

For the Spanish translation used in the study please see supplementary table S2. Emoticons, as shown in supplementary table S2, were shown to study participants only if research staff found that the participant was struggling to understand the response options.

BOX 2 **The psychosocial, socioeconomic, demographic and clinical data collected from study participants at baseline and at 6-months’ follow-up.**

**Table:** 

**The EUROHIS-QOL questionnaire** constitutes eight questions quantifying satisfaction with health, energy, activities of daily living, self, relationships, money, living place and global quality of life (QOL) (see box 1). Each question was answered using a five-point Likert response scale numerically scored *e.g.* 0=“very dissatisfied”, 1=“dissatisfied”; 2=“neither dissatisfied nor satisfied”; 3=“satisfied”; 4=“very satisfied”. As all questions were equally weighted [14], the total score, termed QOL score, was calculated as the sum of the eight questions, with a possible range of 0–32. Higher scores indicated better QOL, a total score of 0=very dissatisfied; 8=dissatisfied; 16=neither dissatisfied nor satisfied; 24=satisfied; and 32=very satisfied in all eight items. There is no defined threshold for good *versus* bad QOL, but a total score of ≤16 suggests on average no satisfaction for any of the eight items, termed “illbeing”. This questionnaire has shown good cross-cultural performance and been validated in numerous languages including Spanish [12]. However, focus groups with 16 research nurses before and after a community pilot evaluation prompted refinements in the Spanish wording and addition of pictures of faces representing the scale, which were used only if individuals struggled to understand the options. These refinements in language are demonstrated in supplementary table S2.
**Baseline data** In addition to the EUROHIS-QOL questionnaire, patients and controls were interviewed using the following: Beck depression inventory II [44], excluding from analysis six potential symptoms of tuberculosis (TB) (appetite, weight loss, health concerns, poor ability to work, tiredness and trouble sleeping) for which higher scores from 0 to 45 indicated low affect and depression; SASCAT (World Bank Shortened and Adapted Social Capital and Perceived Safety Assessment Tool) [45] quantifying the number of perceived sources of emotional support; an adapted 15-item Explanatory Model Interview Catalogue questionnaire [46], for which higher scores from 0 to 45 indicated greater perceived stigma; and a locally validated assessment of basic knowledge of TB disease, treatment, prevention and available services. For all participants, socioeconomic position (poverty) was assessed using completion of secondary education as a proxy measure [47]. Patients were asked a locally validated questionnaire to characterise symptoms and treatment, and their sputum was collected at recruitment for microbiological assessment using sputum smear microscopy, GeneXpert MTB/RIF and thin-layer agar solid culture using the MDR/XDR-TB Colour Test [48].
**Follow-up data** ∼6 months after recruitment and baseline data collection, patients were re-interviewed using the EUROHIS-QOL questionnaire, and asked about treatment and symptom status.
**Treatment outcome** We used Peruvian guidelines to define outcomes. These follow WHO recommendations except for “incomplete treatment” (World Health Organization termed “loss to follow-up”) which Peruvian policy defined as no treatment for >1 month usually due to locally termed “abandonment”. In addition, we included rare patients with treatment suspension or who never commenced treatment [43, 49]. Patients were considered to have adverse treatment outcome if they died during treatment, their treatment failed or they had incomplete treatment. Unknown outcomes (due to missing data or transfer away with unsuccessful follow-up) were excluded from analyses regarding treatment outcome. Recruited patients whose TB diagnosis was later rescinded were excluded in treatment outcome analyses.

### Analysis

Data were analysed using Stata (version 13; StataCorp, College Station, TX, USA). The EUROHIS-QOL was assessed as recommended by the Medical Outcomes Trust Scientific Advisory Committee [17]. Paired analyses were used when comparing patient QOL scores at baseline *versus* follow-up. Multivariable regressions to identify factors associated with QOL and ascertain whether QOL predicted treatment outcome were analysed using backward stepwise selection of variables of interest with p<0.1 in univariable analysis, with robust standard errors and without imputation. QOL clustering at the participant, household and community level was adjusted for by a multilevel random intercepts model. This was done because several characteristics were similar within households, healthcare services varied between communities, and some communities received support aiming to improve TB outcomes from the CRESIPT (Community Randomised Evaluation of a Socio-Economic Intervention to Prevent TB) trial [18]; nongovernmental organisations; and the national TB programme.

### Sample size

The sample size was opportunistic; *post hoc* calculations determined that the study had >90% power at the 95% significance level to detect a 4-point QOL score difference patients *versus* controls, and a 1-point difference contacts *versus* controls.

## Results

**FIGURE 1:**
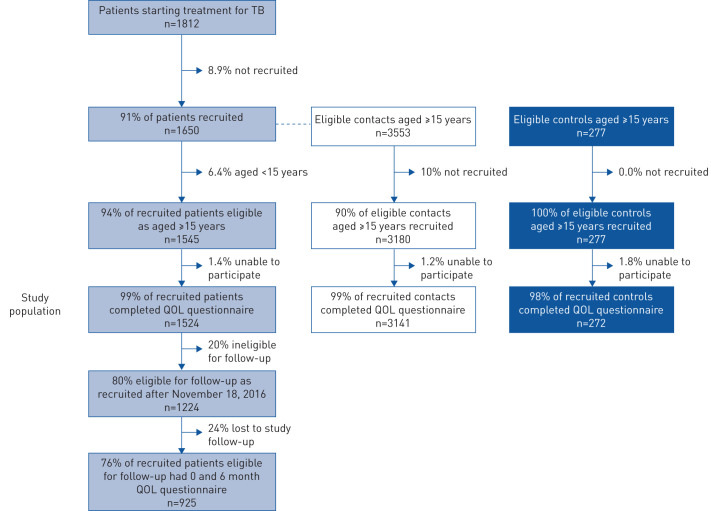
Study flow chart. There were 1650 tuberculosis (TB)-affected households that had a patient starting TB treatment in the participating community health posts (1545 of which had patients who were eligible for the study because they were aged ≥15 years), in which 5885 other people reported spending >6 h·week^−1^ in the fortnight prior to the patient's diagnosis (3553 of whom were eligible for recruitment because they were aged ≥15 years). As controls, 108 randomly selected households were recruited that had 369 inhabitants (277 of whom were eligible for recruitment because they were aged ≥15 years). QOL: quality of life score in the EUROHIS-QOL tool.

Participants' characteristics are summarised in table 1 and figure 1. Data were >97% complete for every variable (table 1).

**TABLE 1:** Participant characteristics

	**Controls**	**Patients**	**Contacts**
**Subjects**	272	1524	3141
**Age years**	35 (24–54)	**31** **(23–46)**	38 (24–52)
**Male % (n)**	38 (102)	**65** **(986)**	39 (1210)
**Incomplete secondary education**	14 (37/271)	16 (236/1516)	**20** **(612/3132)**
**Known HIV seropositivity**	0 (0/272)	**6.2** **(94/1513)**	0.8 (25/3132)
**Self-declared drug use**	2.6 (7/272)	**14** **(207/1512)**	3.1 (98/3136)
**Low affect BDI-II score**	3 (0–5)	**6** **(3–11)**	NA
**Number of emotional supports**	1 (1–2)	**2** **(1–2)**	NA
**Unsafe neighbourhood**	44 (118/270)	**37** **(557/1502)**	NA
**TB-specific**			
Currently has TB	0 (0/272)	**100** **(1524/1524)**	**3.4** **(105/3139)**
Previously had TB	4 (11/271)	**22** **(336/1515)**	**11** **(355/3134)**
TB knowledge correct answers % (IQR)	72 (63–82)	**77** **(73–86)**	NA
Stigma regarding TB EMIC score	30 (23–36)	30 (22–35)	NA
**Patient-specific**			
Pulmonary TB	NA	84 (1278/1516)	NA
Second-line treatment	NA	6.8 (104/1512)	NA
Microbiological confirmation of TB	NA	65 (984/1510)	NA
Number of TB symptoms	NA	7 (5–8)	NA
Duration of TB symptoms months	NA	1.5 (1–3)	NA
Interviewed within 14 days of treatment initiation	NA	86 (1298/1515)	NA

Data are presented as n, median (interquartile range (IQR)) or % (n/N), unless otherwise stated. Denominators are only stated if different from the n number stated for each group. Bold type indicates p<0.05 *versus* the control group. There were only five variables with >1% missing data: low affect (patients: 37 out of 1524), number of emotional supports (controls: three out of 272; patients: 23 out of 1524), unsafe neighbourhood (patients: 22 out of 1524), tuberculosis (TB) knowledge (controls: five out of 272; patients 22 out of 1524) and stigma (controls: four out of 272; patients 30 out of 1524). BDI: Beck Depression Inventory; EMIC: Explanatory Model Interview Catalogue Stigma Questionnaire; NA: not asked.

### QOL assessment

QOL assessment took ∼2–4 min per participant and was completed successfully by 99% (4937 out of 5002) of participants at recruitment (figure 1). For the last 4177 recruitments we noted that only 2.3% of participants used the emoticons shown in supplementary table S2 to help them to answer the questions. The EUROHIS-QOL had good internal consistency between the eight questions (α=0.82) and clustering within households (intraclass coefficient 22%). The parametric distribution of QOL scores is shown in supplementary figure S1. The EUROHIS-QOL construct validity was demonstrated by significant associations with alternative measures of separate QOL domains such as low affect (measured by the Beck Depression Inventory (BDI)-II), emotional support, perceived safety and poverty (table 2).

**TABLE 2:** Univariable regression of associations with quality of life score in the EUROHIS-QOL tool (QOL) separately in controls, patients and contacts

	**Control**		**Patient**		**Contact**	
	**Difference in score (95% CI)**	**p-value**	**Difference in score (95% CI)**	**p-value**	**Difference in score (95% CI)**	**p-value**
**Subjects n**	272		1524		3141	
**QOL**	18.1±4.4		14.2±5.0		17.6±4.2	
**Age (per decade)**	**−0.64 (−0.94–−0.34)**	**<0.0001**	**−0.39 (−0.54–−0.23)**	**<0.0001**	**−0.39 (−0.47–−0.30)**	**<0.0001**
**Female**	−0.45 (−1.4–0.54)	0.4	**−0.90 (−1.4–−0.39)**	**0.001**	**−1.3 (−1.6–−1.0)**	**<0.0001**
**Incomplete secondary education**	**−2.2 (−3.9–−0.36)**	**0.02**	**−1.73 (−2.4–−1.0)**	**<0.0001**	**−1.5 (−1.9–−1.1)**	**<0.0001**
**Known HIV seropositivity**	NC		**−1.4 (−2.6–−0.21)**	**0.02**	**−2.9 (−5.1–−0.62)**	**0.01**
**Self-declared drug use**	0.11 (−2.5–2.7)	0.9	**−2.2 (−2.9–−1.4)**	**<0.0001**	−0.44 (−1.4–0.49)	0.4
**Low affect (BDI-II score)**	**−0.26 (−0.43–−0.09)**	**0.003**	**−0.31 (−0.35–−0.28)**	**<0.0001**	NA	
**Number of emotional supports**	**0.42** **(0.003–0.84)**	**0.05**	**0.60** **(0.38–0.83)**	**<0.0001**	NA	
**Unsafe neighbourhood**	**−1.0 (−2.1–0.01)**	**0.05**	**−1.5 (−2.1–−1.0)**	**<0.0001**	NA	
**TB-specific**						
Currently has TB	NC		NC		**−1.6 (−2.6–−0.55)**	**0.003**
Previously had TB	2.2 (−5.5–1.2)	0.2	**−1.6 (−2.2–−1.1)**	**<0.0001**	−0.45 (−0.95–0.04)	0.07
TB knowledge	NA		**0.02** **(0.004–0.04)**	**0.02**	NA	
Stigma regarding TB (EMIC score)	NA		**−0.10 (−0.13–−0.08)**	**<0.0001**	NA	
**Patient-specific**						
Pulmonary TB disease	NA		−0.54 (−1.3–0.19)	0.2	NA	
Second-line treatment	NA		−0.93 (−1.9–0.06)	0.07	NA	
Microbiological confirmation of TB	NA		**−0.77 (−1.3–−0.23)**	**0.005**	NA	
Number of TB symptoms	NA		**−0.41 (−0.52–−0.31)**	**<0.0001**	NA	
Duration of TB symptoms	NA		**−0.11 (−0.17–−0.05)**	**0.001**	NA	
Days of TB treatment	NA		**0.012** **(0.005–0.02)**	**0.001**	NA	
Patient QOL score	NA		NA		**0.18** **(0.14–0.21)**	**<0.0001**
Carer role to patient	NA		NA		**−1.5 (−1.8–−1.2)**	**<0.0001**

Data are presented as n or mean±sd unless otherwise stated. Bold type indicates p<0.05. BDI: Beck Depression Inventory; TB: tuberculosis; EMIC: Explanatory Model Interview Catalogue Stigma Questionnaire; NC: not calculated as either no-one or everyone had the characteristic (see table 1); NA: not asked.

### Participants

As shown in table 1, 84% (1278 out of 1516) of patients who completed the EUROHIS-QOL had pulmonary TB. At recruitment, 86% (1295 out of 1500) of patients were interviewed within 14 days of treatment, and 6.8% (104 out of 1512) commenced or were about to commence second-line treatment for drug-resistant TB. In addition to patients, 3.4% (105 out of 3139) of contacts were recently diagnosed with TB.

### QOL at recruitment

**FIGURE 2:**
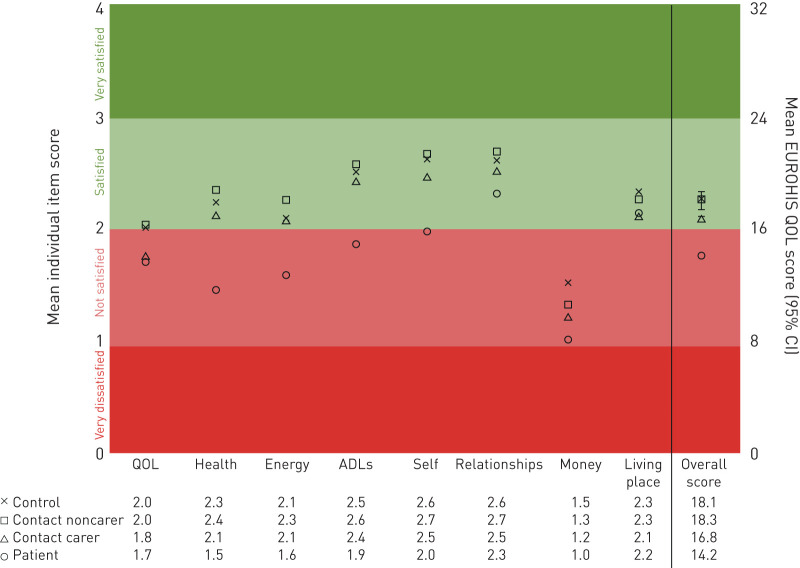
Overall EUROHIS-QOL (quality of life) and individual item score at baseline. The participant groups are controls (n=272); contacts who were not patient carers (n=1765); contacts who were patient carers (n=1376); and patients (n=1524). The right-hand axis shows the EUROHIS-QOL score and left-hand axis shows the individual item score. Both axes are divided as very dissatisfied (EUROHIS-QOL score 0–7 and individual item score 0), not satisfied (EUROHIS-QOL score 8–16 and individual item score 1 or 2), satisfied (EUROHIS-QOL score 17–24 and individual item score 3) and very satisfied (EUROHIS-QOL score 25–32 and individual item score 4). ADL: activities of daily life.

Compared with controls (n=272) or contacts (n=3141), patients (n=1524) had lower QOL overall (table 2 and figure 2). Lower QOL for patients *versus* controls were noted for all eight QOL questions (p≤0.01), especially satisfaction with health, ability to perform activities of daily living, and self. Table 2 demonstrates the factors that impact QOL in control, patients and contact groups separately, and additional analyses of patient-specific factors associated with QOL are shown in supplementary table S1. For patients, the strongest association with QOL was affect measured by BDI-II (R^2^ 20%, coefficient −0.31, p<0.0001). The difference in BDI-II scores for patients *versus* controls was greatest for the question concerning feelings of guilt (difference 31%, 95% CI 26–37%; p<0.0001, data not shown). Contacts had intermediate QOL between patients and controls, with lower scores if they had TB, were considered to be a carer or lived with a patient who also had low QOL (all p<0.0001). Contacts who did not have TB or a caring role had similar QOL to controls (p=0.6).

### Patient follow-up

Patient follow-up was completed in 925 patients, 76% of the eligible population (figure 1). QOL at recruitment was similar between ineligible *versus* eligible patients (supplementary figure S2). However, follow-up QOL assessment was confounded by the impossibility of reassessing patients who had died and some patients who were lost to follow-up (supplementary figure S3). In addition, 266 (29%) patients were still receiving treatment during follow-up. Patients who reported symptom improvement at 6-month follow-up (869 out of 925) had a mean 3.7-point QOL score increase (95% CI 3.3–4.1), whereas those who felt worse (21 out of 925) reported a mean 0.10-point QOL score decrease (95% CI −0.46–0.26).

### Multivariable regression

**FIGURE 3:**
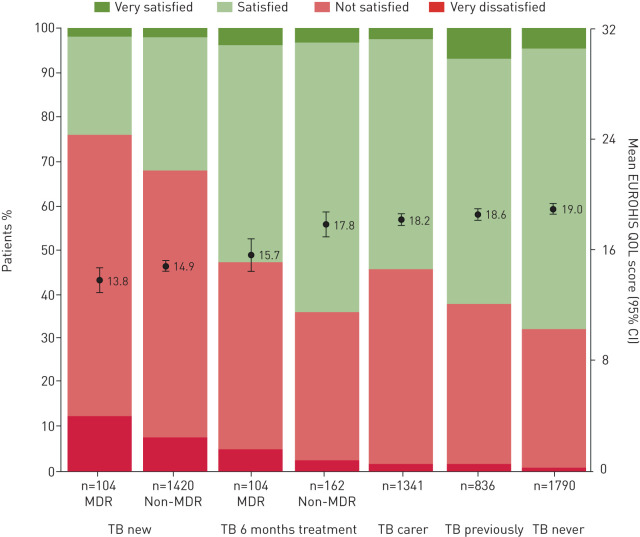
Quality of life (QOL) adjusted for age, sex, and if taking treatment, the days between interview and treatment initiation. Multilevel multivariable analysis was used to adjust the QOL score to that of a 30-year-old male from a typical household and community. MDR: multidrug-resistant; TB new: baseline data for patients newly diagnosed with tuberculosis disease; TB 6 months treatment: data for patients continuing TB treatment at the 6-month follow-up; TB carer: contacts who were the parent or spouse of a patient in their household, and assumed to have a caring role; TB previously: controls and contacts who were not TB carers and reported having received TB treatment previously, and patients at 6-month follow-up who were no longer receiving TB treatment; TB never: controls and contacts who reported never having received TB treatment and were not currently TB carers. The allocation of participants into the aforementioned groups is shown in supplementary figure S4. Circles and error bars show the mean EUROHIS-QOL score (95% CI), and underlying stack plots show the proportion of participants who reported QOL scores 0–7 (very dissatisfied), 8–16 (not satisfied), 17–24 (satisfied) and 25–32 (very satisfied).

Crude comparisons between patients (at recruitment and follow-up), contacts and controls were confounded by several participant characteristics (*e.g.* sex, age, current or previous TB and days of TB treatment) being independently associated with both TB disease (table 1) and QOL (table 2). Therefore, for the following multilevel regression, participants were regrouped according to TB status (supplementary figure S4) and analysis was adjusted for age, sex and if they were taking treatment the days between interview and TB treatment initiation. Contacts with current TB (n=105) were excluded as details of TB disease and treatment were unknown; consequently, 5757 QOL interviews were included in the following analysis (table 3 and figure 3).

**TABLE 3:** Multivariable multilevel model assessing predictors of quality of life in the EUROHIS-QOL tool (QOL) in the study population

	**Adjusted for age, sex and if receiving treatment, the time between interview and treatment initiation**		**Multivariable analysis**	
	**Difference in score (95% CI)**	**p-value**	**Difference in score (95% CI)**	**p-value**
**TB new (MDR)**^#^	**−5.19 (−6.05–−4.34)**	**<0.0001**	**−4.67 (−5.52–−3.81)**	**<0.0001**
**TB new (non-MDR)**^#^	**−4.11 (−4.41–−3.81)**	**<0.0001**	**−3.84 (−4.15–−3.54)**	**<0.0001**
**TB 6 months treatment (MDR)**^¶^	**−3.32 (4.47–−2.17)**	**<0.0001**	**−3.04 (−4.19–−1.89)**	**<0.0001**
**TB 6 months treatment (non-MDR)**^¶^	**−1.17 (−2.06–−0.27)**	**0.01**	**−0.89 (−1.79–0.004)**	**0.05**
**TB carer**^+^	**−0.76 (−1.08–−0.44)**	**<0.0001**	**−0.71 (−1.02–−0.39)**	**<0.0001**
**TB previously**^§^	**−0.39 (−0.74–−0.03)**	**0.03**	−0.2 (−0.55–0.16)	0.3
**TB never**^ƒ^	Reference		Reference	
**Age (for every 10 years)**			**−0.35 (−0.43–−0.27)**	**<0.0001**
**Female**			**−1.08 (−1.31–−0.85)**	**<0.0001**
**Less than secondary education**	**−0.80 (−1.2–−0.44)**	**<0.0001**	**−0.56 (−0.91–−0.22)**	**0.001**
**HIV**	**−3.19 (3.95–−2.43)**	**<0.0001**	**−1.81 (−2.52–−1.08)**	**<0.0001**
**Drug use**	**−2.91 (−3.41–−2.41)**	**<0.0001**	**−1.94 (−2.42–−1.46)**	**<0.0001**
**Days between interview and treatment initiation**^##^			**0.003 (0.0006–0.006)**	**0.02**

The analysis handled clustering at the participant, family and community level using random intercepts. TB: tuberculosis; MDR: multidrug-resistant; NA: not applicable. ^#^: baseline data for patients newly diagnosed with TB disease; ^¶^: data for patients continuing TB treatment at the 6-month follow-up; ^+^: contacts who were the parent or spouse of a patient in their household and assumed to have a caring role; ^§^: controls and contacts who were not TB carers and reported having received TB treatment previously, and patients at 6-month follow-up who were no longer receiving TB treatment; ^ƒ^: controls and contacts who reported never having received TB treatment and were not currently TB carers; ^##^: only calculated for patients who were receiving or were planned to receive TB treatment at the time of interview. The allocation of participants into the aforementioned groups is shown in supplementary figure S4.

#### QOL in newly diagnosed patients

Compared with participants who never had TB, patients newly diagnosed with TB starting non-multidrug-resistant (MDR)-treatment had QOL scores 4.1 points lower (p<0.0001) and 68% had illbeing (QOL score ≤16). Newly diagnosed patients starting MDR treatment had the lowest QOL, with QOL scores 1.1 point less than those starting non-MDR treatment (p=0.01), with less satisfaction in all eight QOL questions and 76% reporting illbeing.

#### QOL in patients receiving treatment at 6-month follow-up

Patients still receiving treatment at follow-up had lower QOL scores than participants who never had TB, with those receiving non-MDR treatment scoring 1.2 points less (p=0.01). Participants receiving MDR treatment at the 6-month follow-up had lower QOL, with scores similar to newly diagnosed patients starting non-MDR treatment (p=0.2).

#### QOL in TB carers

Compared with participants who never had TB and were not carers; contacts who were carers had 0.76 point lower QOL(p<0.0001), mainly due to lower scores for satisfaction with overall QOL, self and living place (p≤0.001). Consequently, 46% of carers reported illbeing.

#### QOL associations

Newly diagnosed TB was the strongest predictor of lower QOL, independent of associations with HIV infection (1.8 points decrease), illicit drug use (1.9 points decrease), female gender (1.1 points decrease), incomplete secondary education (0.56 points decrease) and age (0.35 points per decade decrease) (all p<0.01). After adjusting for these associations, people who had past TB had similar QOL scores to people who never had TB (p=0.3) (figure 3).

#### Patient QOL at baseline and follow-up

**FIGURE 4:**
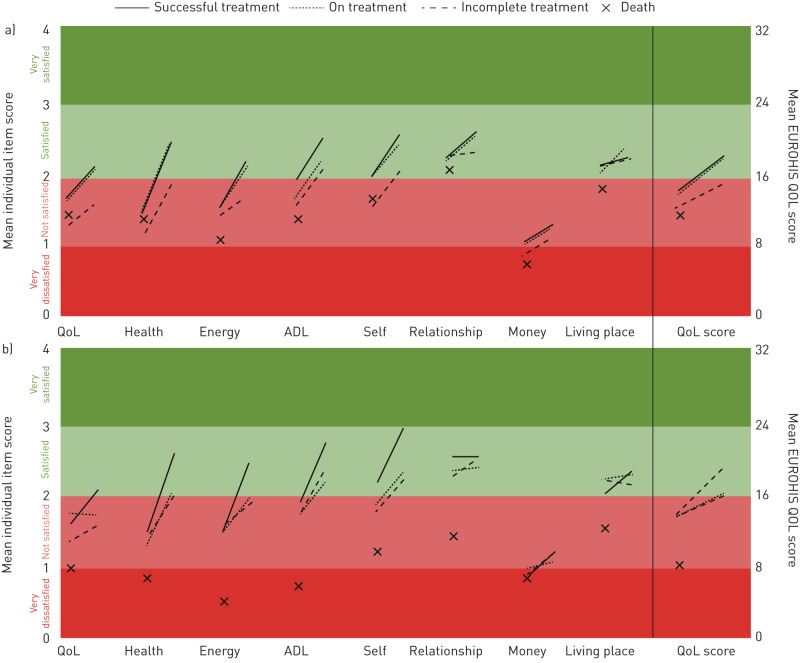
Changes in overall EUROHIS-QOL (quality of life) and individual item score between baseline and 6-month follow-up in patients, stratified by treatment outcome at time of follow-up (n=925). a) patients with non-multidrug-resistant (MDR) tuberculosis and who had successful treatment (n=538), were still receiving treatment (n=162), had incomplete treatment (n=54) or had died prior to follow-up (n=29); b) patients with MDR-TB and who had successful treatment (n=26), were still receiving treatment (n=104), had incomplete treatment (n=24) or had died prior to follow-up (n=9). Patients whose treatment failed (n=5) are not shown.

In 925 patient follow-ups, 157 (21%) were in those who received MDR treatment. QOL at 6 months increased the most for the 564 patients who had successful treatment (mean 3.9 points increase, 95% CI 3.4–4.3; p<0.0001; n=26 received MDR treatment), and QOL scores became similar to those who never had TB (p=0.3) (figure 4). QOL over 6 months improved the least in the 78 patients with incomplete treatment (mean 2.6 points increase, 95% CI 1.4–3.6), regardless of whether they received MDR (n=24) *versus* non-MDR treatment (n=54; p=0.8). Patients still receiving non-MDR treatment at 6 months had similar improvements in QOL as those who had successful treatment (mean 3.8 points increase, 95% CI 2.9–4.6). Whereas patients receiving MDR-TB treatment at the 6-month follow-up had a similar QOL trajectory to those with incomplete treatment (QOL score increase 2.4 points, 95% CI 1.2–3.2), both were still not satisfied with their general QOL, health and energy levels.

#### Patient QOL and treatment outcome

**FIGURE 5:**
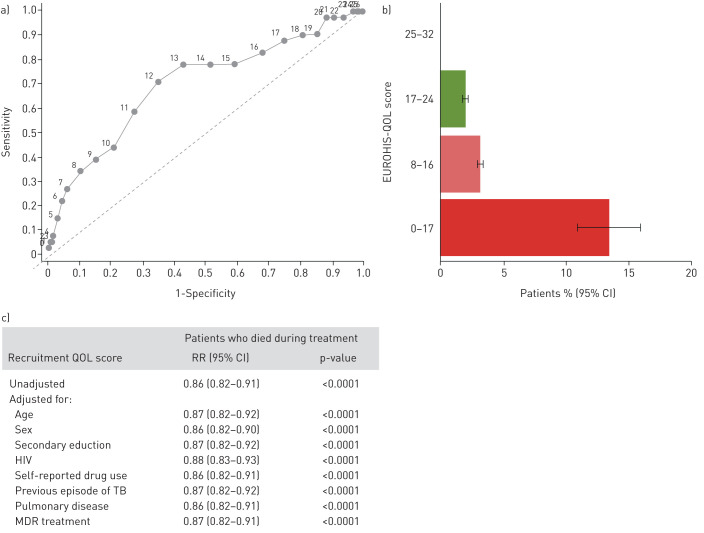
a) Receiver operating curve, b) bar graph and c) adjusted generalised linear model regression of the EUROHIS-QOL score at baseline to predict death. Regression compares patients who died during treatment (n=41) *versus* patients who had successful treatment (n=1008) or were still being treated (n=137). Patients who had incomplete treatment (n=221) or treatment failure (n=9) were excluded. Results for adverse treatment outcome and incomplete treatment are shown in supplementary figures S5 and S6. RR: risk ratio; QOL: quality of life score in the EUROHIS-QOL tool; TB: tuberculosis; MDR: multidrug-resistant.

Treatment outcomes were known for 93% (1416 out of 1524) of patients. Adverse outcome, as described in box 2, occurred in 19% (271 out of 1416) and was more likely for patients with lower QOL at baseline (1.05-fold increased risk for every 1-point decrease QOL score, 95% CI 1.02–1.07; p<0.0001) (supplementary figure S5). Low QOL was better able to predict death (p<0.0001) (figure 5, supplementary figures S5 and S6), which occurred in 2.9% of patients (41 out of 1416, 11 treated for MDR-TB). The area under the receiver operating curve for QOL score predicting death was 0.70 (95% CI 0.61–0.79). Patients with QOL score <13 points at baseline had 4.2-fold (95% CI 2.3–7.6) increased risk of death *versus* those with higher QOL scores. This cut-off predicted death with sensitivity 71% (95% CI 54–84%) and specificity 65% (95% CI 62–68%). Patient risk of death (compared with those with higher initial QOL) was 2.3-fold (95% CI 0.95–5.4) increased if they had illbeing and 4.9-fold (95% CI 2.5–9.7) increased if average QOL was “very dissatisfied” (QOL score <8).

## Discussion

The EUROHIS-QOL eight-item questionnaire was a valid instrument to measure general QOL in TB-affected people. It was successfully completed by almost all participants and demonstrated good reliability, validity and responsiveness as a patient-reported QOL instrument. Patients with TB, especially MDR TB, had lower QOL than community controls, and this was associated not only with TB symptoms, but also with psychological and social dimensions of QOL. Patients with lower QOL at diagnosis were less likely to complete their TB treatment and survive. This study also assessed QOL in patients' household contacts, drawing attention to the negative impact of informal caregiving on QOL.

TB programmes have traditionally focused on treating the infectious TB pathogen and the symptoms it causes. Increasing awareness of the considerable financial consequences of TB disease led to rapid development of metrics to measure TB-related household costs, and the WHO End TB Strategy target to prevent catastrophic costs [5]. In comparison with this recent focus on economic aspects of TB, awareness of the psychosocial burden of TB has neither an agreed metric nor a target. Our findings demonstrate that TB-related QOL can be measured with the simple EUROHIS-QOL questionnaire. It highlights the need to improve TB-related QOL, including the profound dissatisfaction with one's self, relationships and global QOL, potentially worsened by TB-related distress, stigma and isolation. As costing tools are being rolled out to assess the financial burden of TB, we recommend integrating the EUROHIS-QOL eight-item questionnaire to concurrently assess burden on QOL. As recommended by the WHO STAG, this would simultaneously capture all domains of QOL, not just the economic dimension, identifying the more holistic needs of TB-affected households [9].

Patients and carers had less satisfaction with self, with patients expressing high levels of guilt in the BDI-II questionnaire. Fear of exclusion or blame for infecting others may lead to patients feeling guilty, having poor self-esteem, anticipated stigma and worse perceived symptom severity [19, 20]. Longitudinal cohort studies reported that during TB treatment, improvements in mental health took much longer than improvements in physical symptoms [10, 21]. In particular, satisfaction with social and role functioning were at their lowest 1 month after starting TB treatment [22]. In addition, our findings show that QOL was impaired during treatment, especially MDR treatment, and support the WHO recommendations for social support such as education and counselling to improve adherence and treatment completion [23]. We also recommend supplementary socioeconomic support, which can further increase the likelihood of successful treatment, helping to return QOL to the community average [24, 25]. Misinformation and mistreatment from healthcare professionals can contribute to patient dissatisfaction [26]. Within 2 weeks of appropriate treatment the concentration of infectious TB-causing bacteria in sputum usually reduces by 99% and cough frequency more than halves [27, 28]. Therefore, increasing the pragmatism of infection control policies for patients confirmed to be receiving appropriate treatment may improve QOL without increasing TB transmission. Meanwhile, providing training in patients' rights and sharing experiences can challenge discrimination from healthcare workers, while improving working conditions and associated stresses further cultivates compassionate care [29–31].

Low QOL at initiation of TB treatment, and especially scores indicating that a patient was very dissatisfied, was associated with death, adverse treatment outcome and treatment noncompletion. Patients in this study had both PCR- and culture-based drug susceptibility testing and started MDR treatment when drug-resistant TB was suspected or confirmed. Yet, 41 patients died, which the EUROHIS-QOL score predicted. Currently there are no policies in place regarding adjuncts for individuals who are taking seemingly appropriate treatment, but likely to have fatal disease, even though the End TB Strategy aims to have zero TB deaths by 2030 [5]. Impairment of QOL associated with TB defines important multisystem ill health. The EUROHIS-QOL tool was responsive to changes in clinical state and suggested that QOL completely recovered after successfully completing treatment. This contrasts with a study in which patients who had completed TB treatment had lower QOL than healthy controls [32]. Lung destruction due to TB is an important contributor to poor QOL, and pulmonary rehabilitation programmes have been shown to improve lung function and QOL in TB-affected people [33, 34]. Thus, the EUROHIS-QOL may have a role in helping to identify who should be offered enhanced care aiming to reduce the risk of mortality.

Patients' spouses and parents, who were assumed to have a caregiving role, had significantly lower QOL than other contacts, independent of confounding differences in age, sex and clustering of data within households. Additionally, lower patient QOL was associated with lower QOL in contacts, which may be explained by socioeconomic risks shared by patients and their caregivers, but also the socioeconomic stress of caring for someone with TB. Furthermore, caregivers may “absorb” some of the stigma, emotional and financial costs of TB [35–37]. Stress during informal caregiving has been associated with increased all-cause mortality, possibly due to allostatic load and cellular ageing [38–40]. However, these studies were in the context of geriatrics, oncology and paediatrics and reviews from these specialities have shown that involving caregivers in patients' management plans, educational activities and psychosocial support can alleviate caregiver burden [41]. This may be feasible and appropriate for TB programmes.

A limitation of this study is that all QOL questionnaires including the EUROHIS-QOL are inevitably subjective, so prone to response bias including social desirability. This bias was reduced by ensuring privacy, confidentiality and data collection by researchers who were independent of the healthcare system. There is no gold standard method to objectively assess general QOL. However, good correlation of the EUROHIS-QOL responses with other objective determinants of QOL implies that response bias did not undermine our findings. A strength of our study was that we were able to disaggregate patient QOL scores depending on TB treatment scheme and outcome. However, this was limited by the reduced number of patients eligible for follow-up who completed the 6-month interview, partly due to the impossibility of assessing follow-up QOL for people who had died, and difficulty locating people who had incomplete TB treatment. The generalisability of our findings is supported by the diversity of our study setting including 15 peri-urban shantytowns and 17 urban communities; and supports previous studies of the psychosocial costs of TB in other continents and social contexts [10, 16, 42]. Another limitation is that we did not study preventative treatment, which could have influenced QOL. However, this could only have affected a tiny proportion of our study population because chemoprophylaxis was probably started after the recruitment interview when we assessed QOL (at the time the index patient commenced treatment); we only assessed QOL in people aged ≥15 years, and in this age group in Peru, chemoprophylaxis was only recommended for people with HIV infection and 15–19-year-olds who were known to be tuberculin skin test-positive close contacts of patients with isoniazid-susceptible TB [43]; and even in this group, there is a very low uptake of chemoprophylaxis [24]. A further limitation is that treatment for MDR-TB in Peru took >2 years at the time of the study, so QOL after MDR-TB treatment could not be evaluated in this study. The number of control households was opportunistic, not determined by sample size calculations, but the significant differences between controls and other participant groups suggests that this sample was generally sufficient.

In conclusion, the brief EUROHIS-QOL eight-item questionnaire meaningfully assessed QOL in TB-affected people and may be used to assess general QOL associated with TB disease in future research and practice. We recommend research to evaluate the EUROHIS-QOL with TB-affected households in diverse settings and trials to assess whether integrating the EUROHIS-QOL as a part of TB care can improve the quality of holistic care for members of TB-affected households. Our data show the profound psychosocioeconomic burden of TB for patients and their caregivers, and that low patient QOL predicts adverse treatment outcomes, especially death. These findings support the use of the EUROHIS-QOL to identify and quantify the holistic needs of people living with TB, potentially guiding patient-centred care addressing these multidisciplinary needs.

## Supplementary material

10.1183/13993003.00495-2019.Supp1 **Please note:** supplementary material is not edited by the Editorial Office, and is uploaded as it has been supplied by the author.
Supplementary material ERJ-00495-2019.SUPPLEMENT

## Shareable PDF

10.1183/13993003.00495-2019.Shareable1 This one-page PDF can be shared freely online.
Shareable PDF ERJ-00495-2019.Shareable

## Supplementary Material

ERJ-00495-2019.SUPPLEMENT.pdf

ERJ-00495-2019.Shareable.pdf
