# Supplementary material for: Quality of life, tuberculosis and treatment outcome; a case–control and nested cohort study
Source: Eur Respir J. 2020 Aug 6;56(2):1900495. doi: 10.1183/13993003.00495-2019 (PMC7406858; doi:10.1183/13993003.00495-2019)
Supplement: Supplementary file 1 [file ERJ-00495-2019.SUPPLEMENT.pdf]

SUPPLEMENTARY TABLE S1. Multivariable regression to predict quality of life (QOL) EUROHIS-QOL scores in patients with tuberculosis (TB). Multivariate model 1 had the best fit with R^2=27%. Multivariate model 2 excluded covariates (EC) of QOL (negative affect, number of emotional supports and unsafe neighbourhood) with R^2=16%. For the multivariable analyses, variables that did not contribute (NC) significantly to the model were removed in a backward stepwise manner. Note. CI=confidence interval.

	Univariate		Multivariate model 1		Multivariate model 2 without covariates of QOL	
	Difference in score (95%CI)	p value	Difference in score (95%CI)	p value	Difference in score (95%CI)	p value
Age (per decade)	-0.39 (-0.54, -0.23)	<0.0001	-0.24 (-0.39, -0.11)	0.001	-0.21 (-0.37, -0.05)	0.01
Female	-0.90 (-1.4, -0.39)	0.001	-0.79 (-1.3, -0.31)	0.001	-1.5 (-2.0, -0.97)	<0.0001
Incomplete secondary education	-1.73 (-2.4, -1.0)	<0.0001	NC		NC	
Known HIV seropositivity	-1.4 (-2.6, -0.21)	0.02	NC		-1.6 (-2.7, -0.41)	0.008
Self-declared drug use	-2.2 (-2.9, -1.4)	<0.0001	-1.4 (-2.1, -0.68)	<0.0001	-2.2 (-2.9, -1.4)	<0.0001
Low affect (BDI-II score)	-0.31 (-0.35, -0.28)	<0.0001	-0.24 (-0.27, -0.20)	<0.0001	EC	
Number of emotional supports	0.60 (0.38, 0.83)	<0.0001	0.30 (0.11, 0.51)	0.003	EC	
Unsafe neighborhood	-1.5 (-2.1, -1.0)	<0.0001	-0.96 (-1.42, -0.50)	<0.0001	EC	
TB disease specific						
Previously had TB	-1.6 (-2.2, -1.1)	<0.0001	-0.94 (-1.5, -0.40)	0.001	-1.4 (-1.9, -0.77)	<0.0001
TB knowledge	0.02 (0.004, 0.04)	0.02	NC		0.029 (0.01, 0.049)	0.005
TB stigma (EMIC score)	-0.10 (-0.13, -0.08)	<0.0001	-0.058 (-0.082, -0.033)	<0.0001	-0.10 (-0.13, -0.07)	<0.0001
Pulmonary disease	-0.54 (-1.3, 0.19)	0.2	NC		NC	
Second line therapy	-0.93 (-1.9, 0.06)	0.07	NC		NC	
Microbiological confirmation	-0.77 (-1.3, -0.23)	0.005	NC		NC	

Number of symptoms	-0.41 (-0.52, -0.31)	<0.0001	-0.25 (-0.34, -0.15)	<0.0001	-0.36 (-0.46, -0.26)	<0.0001
Duration of symptoms	-0.11 (-0.17, -0.05)	0.001	NC		-0.066 (-0.12, -0.013)	0.02
Interviewed after >14 days of TB therapy	1.0 (0.31, 1.8)	0.005	0.71 (0.011, 1.4)	0.05	0.93 (0.17, 1.7)	0.02

SUPPLEMENTARY TABLE S2. The EUROHIS-QOL wellbeing tool in the Spanish translation that was used in this study, the original Spanish in sentences that were adapted for cross-cultural harmonization. Note: the pictures below the table were added and shown to study participants only if research staff found that the participant was struggling to understand the responses shown in the box.

SPANISH TRANSLATION USED IN STUDY		ORIGINAL SPANISH TRANSLATION	
Por favor, escuche las preguntas, valore sus sentimientos y escoja la mejor opción para usted. Le pedimos que sus respuestas estén en relación a su vida en las últimas dos semanas.			
**Preguntas**	**Opciones**	**Preguntas**	**Opciones**
1. ¿Cómo calificaría su calidad de vida?	**A**. Muy mala<br>**B**. Regular<br>**C**. Normal<br>**D**. Buena<br>**E.** Muy buena		**D**. Bastante buena
2. ¿Cuán satisfecho/a está con su salud?	**A.** Muy insatisfecho/a<br>**B.** Un poco insatisfecho/a<br>**C.** Ni satisfecho ni insatisfecho<br>**D.** Satisfecho/a<br>**E.** Muy satisfecho/a	2. ¿Cómo de satisfecho/a está con su salud?	**C.** Lo normal<br>**D.** Bastante satisfecho/a
3. ¿Tiene energía suficiente para su vida diaria?	**A.** Nada<br>**B.** Un poco<br>**C**. Lo suficiente<br>**D.** Bastante<br>**E.** Totalmente		**C**. Lo normal
4. ¿Cuán satisfecho/a está con su habilidad para realizar sus actividades de la vida diaria?	**A.** Muy insatisfecho/a<br>**B.** Un poco insatisfecho/a<br>**C.** Ni satisfecho ni insatisfecho<br>**D.** Bastante satisfecho/a<br>**E.** Muy satisfecho/a	4. ¿Cómo de satisfecho/a está con su habilidad para realizar sus actividades de la vida diaria?	**B.** Poco<br>**C.** Lo normal
5. ¿Cuán satisfecho/a está de sí mismo?	**A.** Muy insatisfecho/a<br>**B.** Un poco insatisfecho/a<br>**C.** Ni satisfecho ni insatisfecho<br>**D.** Bastante satisfecho/a<br>**E.** Muy satisfecho/a		**B.** Poco<br>**C.** Lo normal
6. ¿Cuán satisfecho/a está con sus relaciones personales (entorno social)?	**A.** Muy insatisfecho/a<br>**B.** Un poco insatisfecho/a<br>**C.** Ni satisfecho ni insatisfecho<br>**D.** Bastante satisfecho/a<br>**E.** Muy satisfecho/a	6. ¿Cómo de satisfecho/a está con sus relaciones personales?	**B.** Poco<br>**C.** Lo normal
7. ¿Tiene suficiente dinero para cubrir sus necesidades?	**A.** Nada<br>**B.** Un poco<br>**C.** Lo suficiente		**C.** Lo normal

	**D.** Bastante<br>**E.** Totalmente		
8. ¿Cuán satisfecho/a está de las condiciones del lugar donde vive?	**A.** Muy insatisfecho/a<br>**B.** Un poco insatisfecho/a<br>**C.** Ni satisfecho ni insatisfecho<br>**D.** Bastante satisfecho/a<br>**E.** Muy satisfecho/a	8. ¿Cómo de satisfecho/a está de las condiciones del lugar donde vive?	**B.** Poco<br>**C.** Lo normal
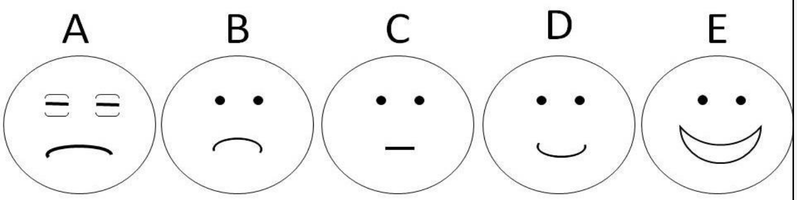			

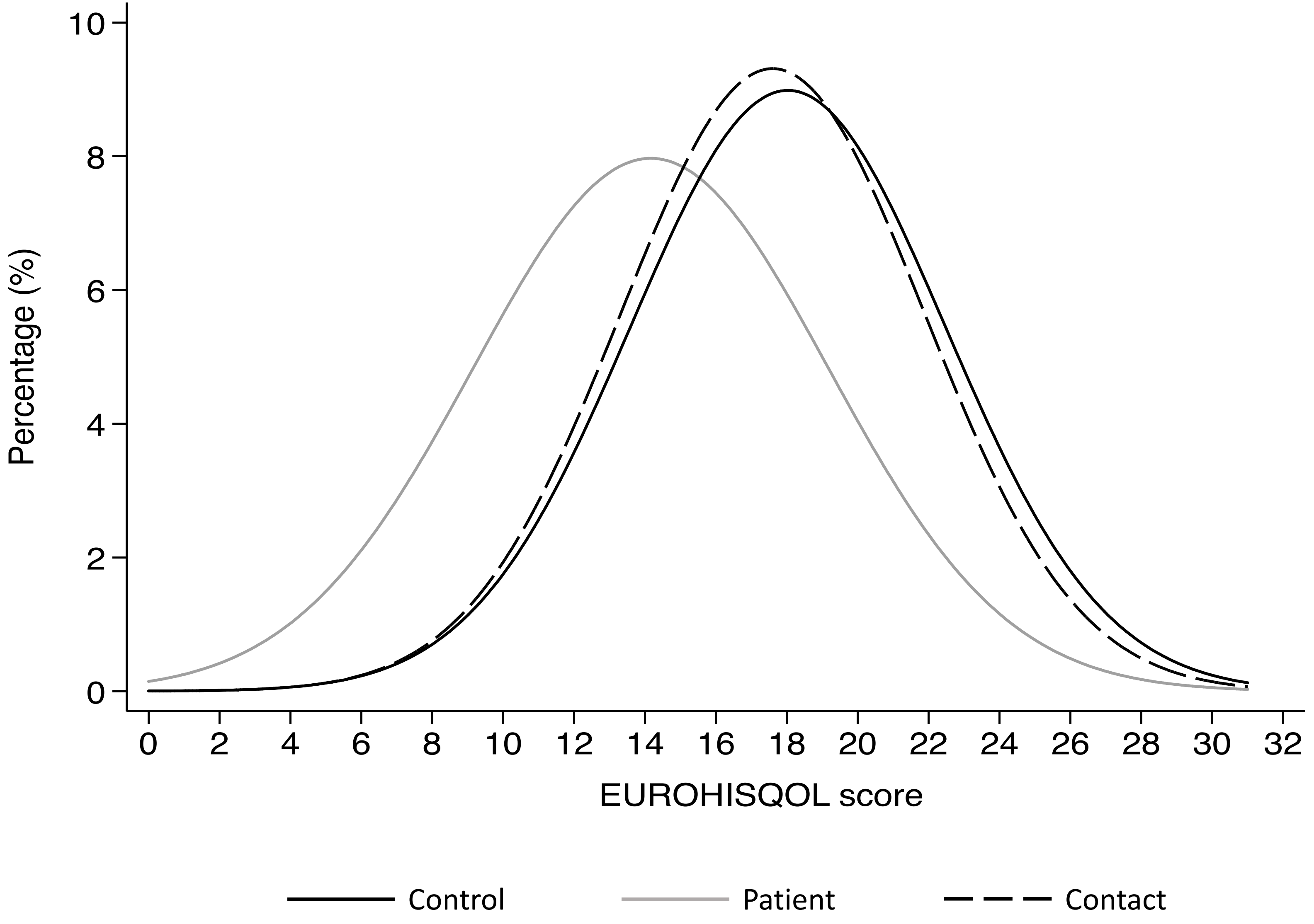

SUPPLEMENTARY FIGURE S1. Distribution of quality of life (QOL) EUROHIS-QOL scores at baseline in controls, patients and contacts.

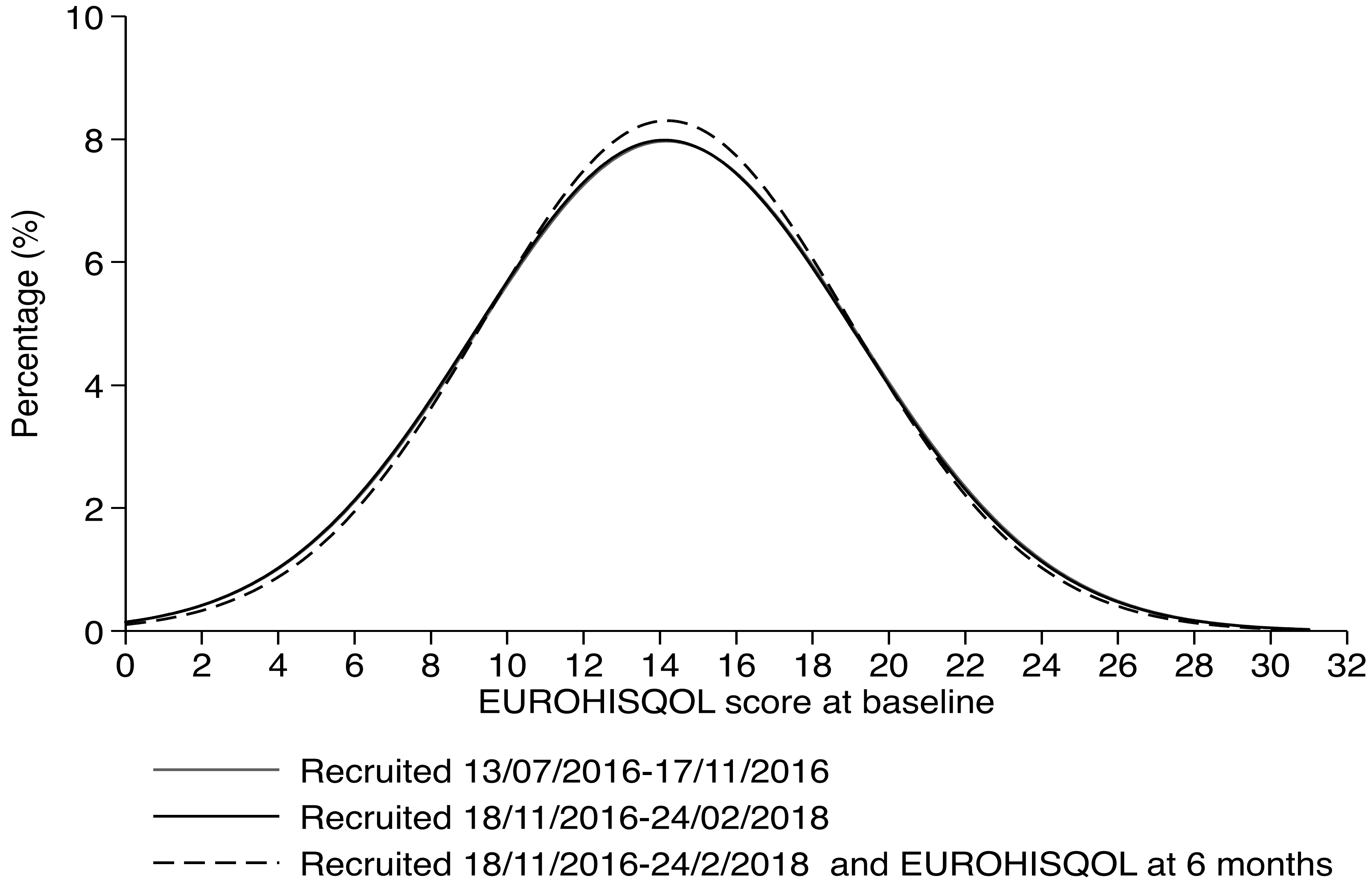

SUPPLEMENTARY FIGURE S2. Distribution of quality of life (QOL) EUROHIS-QOL scores at baseline in patients ineligible for 6 month follow-up (N=300), patients eligible for 6 month follow-up (N=1224), and patients eligible for 6 month follow-up with successful follow-up (N=925).

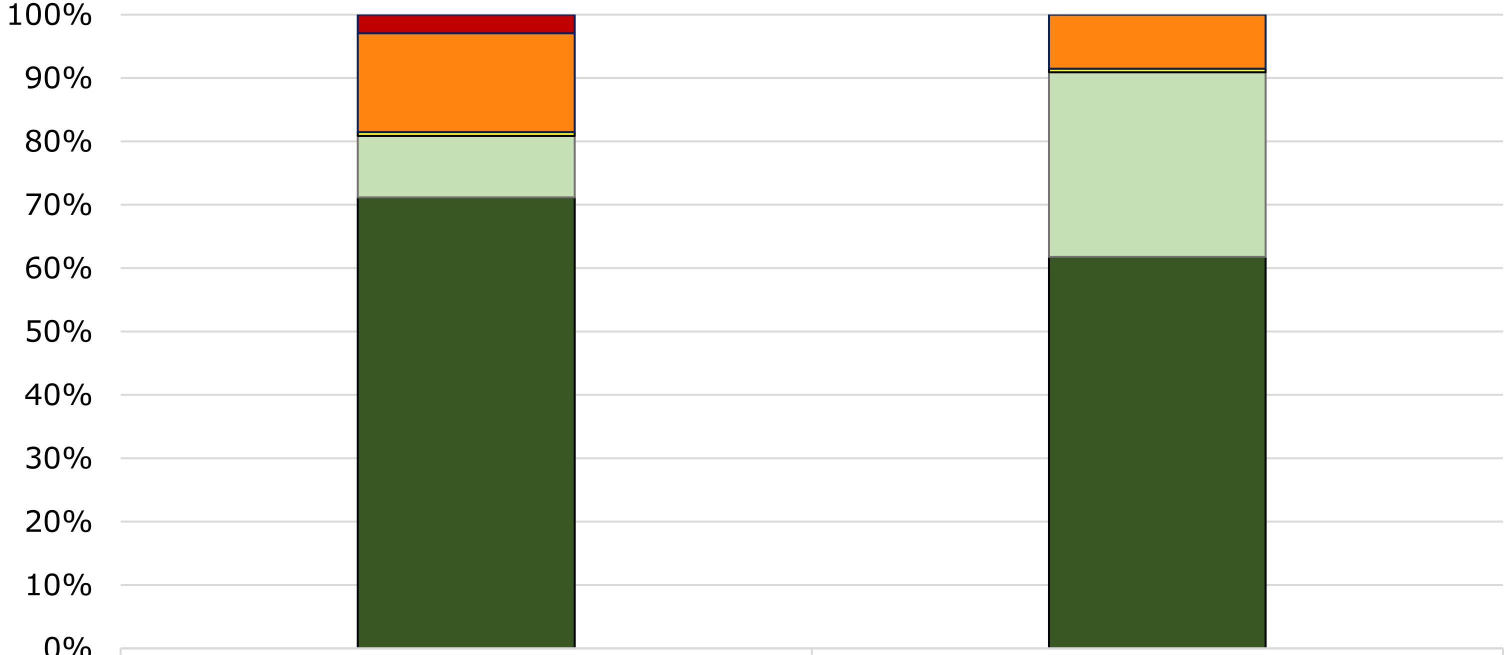

	Patients with baseline QOL	Patients with 6 month follow-up QOL
Died	41	0
Treatment incompletion	221	78
Failed	9	5
On treatment	137	266
Successful	1008	564

SUPPLEMENTARY FIGURE S3. Treatment outcome in patients with known outcome data who completed quality of life (QOL) EUROHIS-QOL questionnaires at baseline and in those with the repeated QOL data at 6-month follow-up. Note patients whose treatment outcome was unknown due to transfer of care or missing data (n=101 interviews at baseline, n=10 interviews at follow-up), or had their diagnosis rescinded after recruitment (n=7 interviews at baseline, n=2 interviews at follow-up), are not shown in this figure.

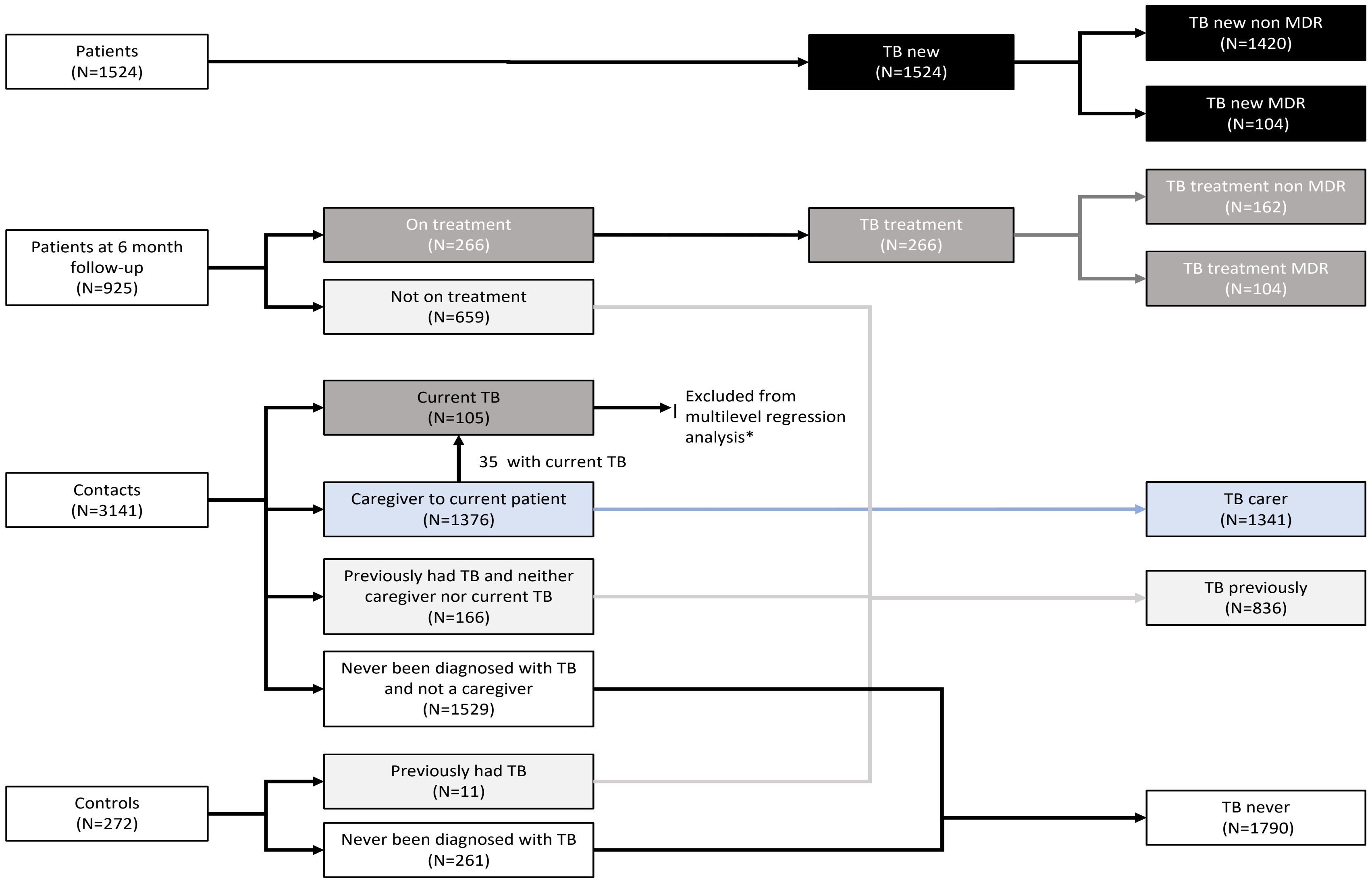

SUPPLEMENTARY FIGURE S4. Patient allocation according to treatment status

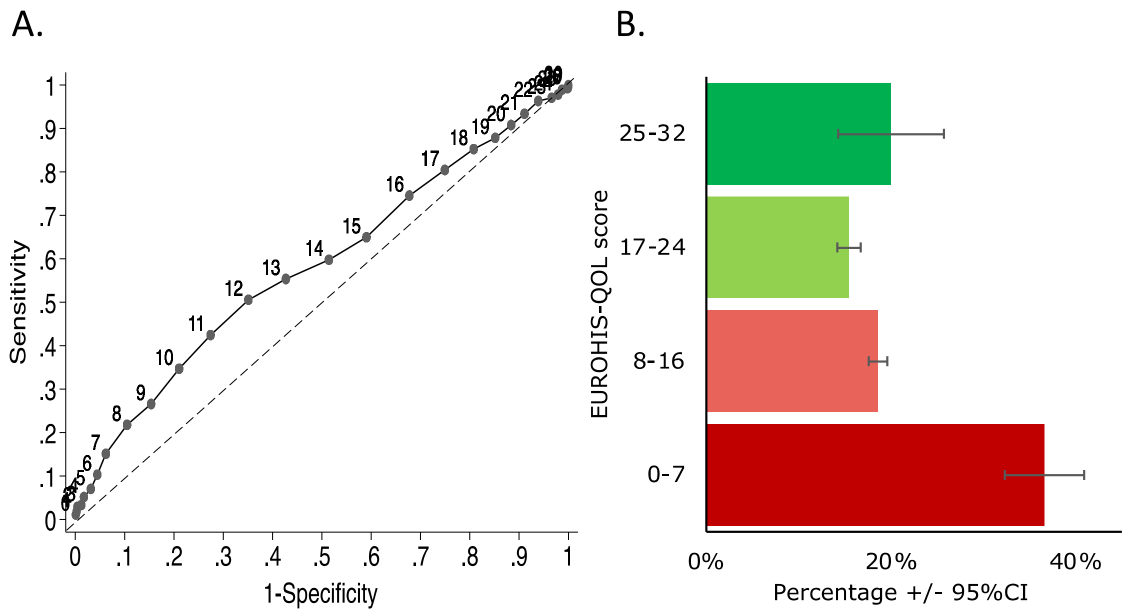

C.

Recruitment QOL score	Adverse treatment outcome RR	95% CI	p value
Unadjusted	0.95	0.93, 0.98	<0.0001
Adjusted for:			
Age	0.95	0.92, 0.97	<0.0001
Gender	0.95	0.92, 0.97	<0.0001
Secondary education	0.95	0.93, 0.98	<0.0001
HIV	0.95	0.93, 0.98	<0.0001
Self reported drug use	0.97	0.94, 0.99	0.009
Previous episode of TB	0.96	0.94, 0.99	0.005
Pulmonary disease	0.95	0.92, 0.98	<0.0001
MDR treatment	0.95	0.92, 0.98	0.001

SUPPLEMENTARY FIGURE S5. (A) Receiver operating curve, (B) bar graph, and (C) adjusted GLM regression of the EUROHIS-QOL score at baseline to predict adverse treatment outcome. Regression compares patients who had an adverse treatment outcome during treatment (n=271), versus patients who had successful treatment (n=1008) or were still being treated (n=137). The area under the curve was 0.54 (95%CI=0.54-0.62). Results for death and incomplete treatment are shown in Figure 5 and SUPPLEMENTARY Figure 6. Low QOL at the time patients with TB commenced therapy, and especially severe illbeing (EUROHIS-QOL score=0-7) indicating being on average very dissatisfied in all 8 QOL questions predicted death, adverse treatment outcome and treatment non-completion. Interestingly, patients with the highest QOL indicating that they were more than satisfied in the 8 QOL questions also tended to be associated with a slightly higher risk of treatment non-completion. This trend supports our qualitative impression from the ongoing CRESIPT trial[19] that patients are at risk of not completing their therapy not only if they are suffering high psycho-socio-economic challenges, but paradoxically also if their symptoms are mild and their socioeconomic situation is unusually favourable. These findings and observations warrant prospective

study. Note: RR=risk ratio, CI=confidence interval; QOL=quality of life score in the EUROHIS-QOL tool.

A. B.

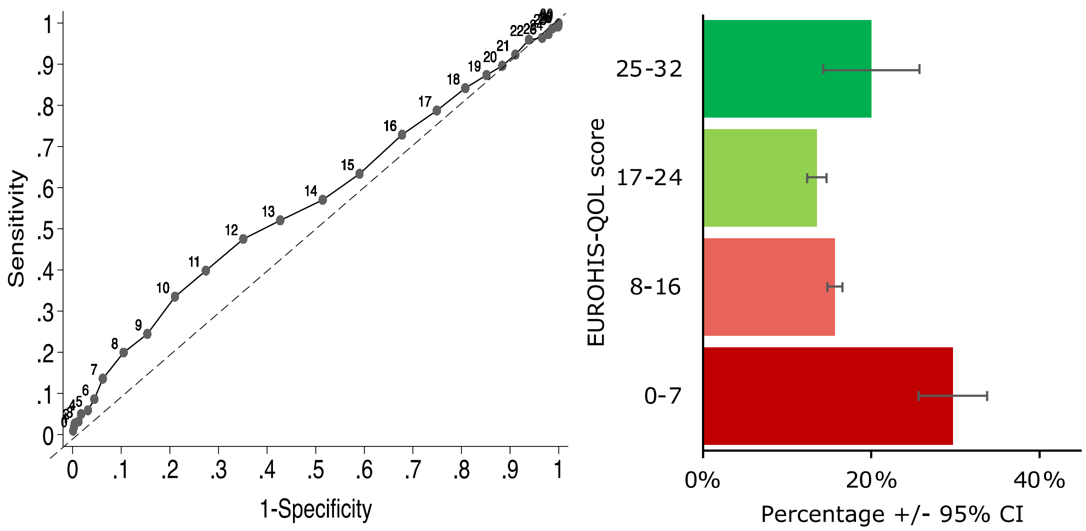

C.

Recruitment QOL score	Incomplete treatment RR	95% CI	p value
Unadjusted	0.96	0.93, 0.99	0.02
Adjusted for:			
Age	0.95	0.92, 0.99	0.006
Gender	0.95	0.92, 0.98	0.003
Secondary education	0.96	0.93, 0.99	0.01
HIV	0.96	0.93, 0.99	0.01
Self reported drug use	0.98	0.95, 1.00	0.1
Previous episode of TB	0.97	0.94, 1.00	0.09
Pulmonary disease	0.96	0.93, 0.99	0.02
MDR treatment	0.96	0.93, 0.99	0.02

SUPPLEMENTARY FIGURE S6. (A) Receiver operating curve, (B) bar graph, and (C) adjusted GLM regression of the EUROHIS-QOL score at baseline to predict incomplete treatment. Regression compares patients who had incomplete treatment (n=221), versus patients who had successful treatment (n=1008) or were still being treated (n=137). The area under the curve was 0.56 (95%CI=0.52-0.61). Results for death and adverse treatment outcome are shown in Figure 5 and SUPPLEMENTARY Figure 5. Low QOL at the time patients with TB commenced therapy, and especially severe illbeing (EUROHIS-QOL score=0-7) indicating being on average very dissatisfied in all 8 QOL questions predicted death, adverse treatment outcome and treatment non-completion. Interestingly, patients with the highest QOL indicating that they were more than satisfied in the 8 QOL questions also tended to be associated with a slightly higher risk of treatment non-completion. This trend supports our qualitative impression from the ongoing CRESIPT trial[19] that patients are at risk of not completing their therapy not only if they are suffering high psycho-socio-economic challenges, but paradoxically also if their symptoms are mild and their socioeconomic situation is unusually favourable. These findings and observations warrant prospective study. Note: RR=risk ratio, CI=confidence interval; QOL=quality of life score in the EUROHIS-QOL tool.
