# Supplementary material for: Quality of life, tuberculosis and treatment outcome; a case–control and nested cohort study
Source: Eur Respir J. 2020 Aug 6;56(2):1900495. doi: 10.1183/13993003.00495-2019 (PMC7406858; doi:10.1183/13993003.00495-2019)
Supplement: Supplementary file 2 [file ERJ-00495-2019.Shareable.pdf]

SHAREABLE PDF

Quality of life, tuberculosis and treatment outcome; a case–control and nested cohort study

Sumona Datta[1,2,3,4], Robert H. Gilman [4], Rosario Montoya [2,3], Luz Quevedo Cruz[2,3], Teresa Valencia[2,3], Doug Huff[1,2,3], Matthew J. Saunders [1,2,3] and Carlton A. Evans[1,2,3]

Affiliations: [1]Dept of Infectious Disease, Imperial College London, London, UK. [2]IFHAD: Innovation for Health and Development, Laboratory for Research and Development, Universidad Peruana Cayetano Heredia, Lima, Peru. [3]IPSYD: Innovacion Por la Salud Y el Desarollo, Asociación Benéfica Prisma, Lima, Peru. [4]Dept of International Health, Johns Hopkins Bloomberg School of Public Health, Baltimore, MD, USA.

Correspondence: Sumona Datta, Honorary Specialist Registrar, Section of Adult Infectious Disease, Imperial College London Hammersmith Hospital Campus, Commonwealth Building level 8, 150 Du Cane Road, London W12 0NN, UK. E-mail: sumona.datta@ifhad.org

@ERSpublications
The brief EUROHIS quality-of-life questionnaire can be applied to assist in providing holistic, personalised care to TB-affected families, and guide multisystem interventions required to improve their wellbeing and TB treatment outcome https://bit.ly/2VhDKFM

Cite this article as: Datta S, Gilman RH, Montoya R, *et al.* Quality of life, tuberculosis and treatment outcome; a case–control and nested cohort study. *Eur Respir J* 2020; 56: 1900495 [https://doi.org/10.1183/13993003.00495-2019].

This single-page version can be shared freely online.

ABSTRACT
Background: Global tuberculosis policy increasingly emphasises broad tuberculosis impacts and highlights the lack of evidence concerning tuberculosis-related quality of life (QOL).
Methods: Participants were recruited in 32 Peruvian communities between July 13, 2016 and February 24, 2018 and followed-up until November 8, 2019. Inclusion criteria were age ⩾15 years for "patients" (n=1545) starting treatment for tuberculosis disease in health centres; "contacts" (n=3180) who shared a patient's household for ⩾6 $h \cdot week^{-1}$; and randomly selected "controls" (n=277). The EUROHIS-QOL questionnaire quantified satisfaction with QOL, health, energy, activities of daily living (ADL), self, relationships, money and living place.
Findings: Newly diagnosed tuberculosis was most strongly associated with lower QOL scores ($p<0.001$). Patients initially had lower QOL than controls for all EUROHIS-QOL questions ($p \leqslant 0.01$), especially concerning health, ADL and self. Lower initial QOL in patients predicted adverse treatment outcomes and scores <13 points had 4.2-fold (95% CI 2.3–7.6) increased risk of death *versus* those with higher QOL scores (both $p<0.001$). Patient QOL was re-assessed 6 months later, and for patients with successful treatment QOL became similar to participants who had never had tuberculosis, whereas patients who did not complete treatment continued to have low QOL ($p<0.001$). Multidrug-resistant tuberculosis was associated with lower QOL before and during treatment (both $p<0.001$). Contacts had lower QOL if they lived with a patient who had low QOL score ($p<0.0001$) or were a caregiver for the patient ($p<0.001$).
Conclusions: Tuberculosis was associated with impaired psychosocioeconomic QOL which recovered with successful treatment. Low QOL scores predicted adverse treatment outcome. This brief EUROHIS-QOL eight-item questionnaire quantified the holistic needs of tuberculosis-affected people, potentially guiding patient-centred care.

Copyright ©ERS 2020. This version is distributed under the terms of the Creative Commons Attribution Licence 4.0.
